# Artificial Intelligence and Machine Learning Models for Predicting Drug-Induced Kidney Injury in Small Molecules

**DOI:** 10.3390/ph17111550

**Published:** 2024-11-19

**Authors:** Mohan Rao, Vahid Nassiri, Sanjay Srivastava, Amy Yang, Satjit Brar, Eric McDuffie, Clifford Sachs

**Affiliations:** 1 Preclinical and Clinical Pharmacology and Chemistry, Neurocrine Biosciences, San Diego, CA 92130, USAcsachs@neurocrine.com (C.S.); 2 Open Analytics NV, Jupiterstraat 20, 2600 Antwerp, Belgium; vahid.nassiri@openanalytics.eu

**Keywords:** off-target interactions, machine learning, artificial intelligence, cheminformatics, drug induced kidney injury, computational toxicology

## Abstract

Background/Objectives: Drug-Induced Kidney Injury (DIKI) presents a significant challenge in drug development, often leading to clinical-stage failures. The early prediction of DIKI risk can improve drug safety and development efficiency. Existing models tend to focus on physicochemical properties alone, often overlooking drug–target interactions crucial for DIKI. This study introduces an AI/ML (artificial intelligence/machine learning) model that integrates both physicochemical properties and off-target interactions to enhance DIKI prediction. Methods: We compiled a dataset of 360 FDA-classified compounds (231 non-nephrotoxic and 129 nephrotoxic) and predicted 6064 off-target interactions, 59% of which were validated in vitro. We also calculated 55 physicochemical properties for these compounds. Machine learning (ML) models were developed using four algorithms: Ridge Logistic Regression (RLR), Support Vector Machine (SVM), Random Forest (RF), and Neural Network (NN). These models were then combined into an ensemble model for enhanced performance. Results: The ensemble model achieved an ROC-AUC of 0.86, with a sensitivity and specificity of 0.79 and 0.78, respectively. The key predictive features included 38 off-target interactions and physicochemical properties such as the number of metabolites, polar surface area (PSA), pKa, and fraction of Sp3-hybridized carbons (fsp3). These features effectively distinguished DIKI from non-DIKI compounds. Conclusions: The integrated model, which combines both physicochemical properties and off-target interaction data, significantly improved DIKI prediction accuracy compared to models that rely on either data type alone. This AI/ML model provides a promising early screening tool for identifying compounds with lower DIKI risk, facilitating safer drug development.

## 1. Introduction

Drug-Induced Kidney Injury (DIKI) can result in acute kidney injury (AKI), chronic kidney disease (CKD), or end-stage renal failure [1]. AKI is currently defined as an absolute increase in serum creatinine by 0.3 mg/dL or a relative increase of 50% over 48 h [2]. DIKI accounts for 14–26% of AKI cases in adults and 16% in children, playing a significant role in the incidence of kidney injuries [1,3,4,5]. Beyond its direct impact on patient health, DIKI is a major challenge in drug development, contributing to 8–9% of preclinical and clinical failures across therapeutic areas [6,7]. The public health implications for DIKI are substantial, affecting patients and causing drug attrition during both development studies and post-marketing [3,4,8,9].

Several renal safety biomarkers have been identified [10,11]. Various regulatory agencies like the United States’ Food and Drug Administration (FDA), European Medicines Agency (EMA), and Japan’s Pharmaceuticals and Medical Devices Agency (PMDA) have recognized urinary proteins as promising indicators for the early detection of DIKI. The qualified nephrotoxicity biomarkers include kidney injury molecule-1 (KIM-1) [12], albumin (ALB) [13], clusterin (CLU) [11,12,13], trefoil factor-3 (TFF-3) [14], total protein [15], cystatin C (CysC) [15], β2-microglobulin (B2M) [15], renal papillary antigen 1 (RPA-1) [16], and neutrophil gelatinase-associated lipocalin (NGAL) [17] for limited use in nonclinical studies and/or case by case in human clinical studies to help guide renal safety assessment. Urinary KIM-1 and CLU are particularly effective for the early detection of DIKI. Urinary albumin is effective for acute, subacute, subchronic, and chronic DIKI. Conversely, traditional nephrotoxicity indicators like serum creatinine (sCr) and/or blood urea nitrogen (BUN) are often less sensitive for detecting incidences of DIKI. However, a deeper understanding of how these biomarkers are modulated by on- and off-target interactions relative to compound-induced pathway-specific outcomes is crucial for improving DIKI prediction. A critical factor in predicting DIKI risk is the clinical dose, as the accuracy of models often relies on clinical maximum plasma concentration (Cmax) or clinical area under the plasma concentration (AUC) data [18]. However, in early drug discovery, accurate clinical dose estimates are typically unavailable, limiting the reliability of initial DIKI risk assessments [19,20]. The mechanisms driving DIKI, particularly those related to off-target interactions in the kidney, are not well understood. In addition, since animal models may not adequately predict adverse effects on human kidneys, particularly if these are driven by species-specific kinetics and/or patient-population-related mechanisms, there remains a gap in the development of next-generation in silico approaches that do not rely on robust human datasets.

At present, various in silico models exist for predicting nephrotoxicity [21,22,23,24,25]. These models typically leverage phenotypic cell-based assays or chemical descriptors derived from natural products or in vitro cellular perturbation data. However, to our knowledge, none of the published models have incorporated a combined approach using chemical descriptors with biological interactions to enhance the capabilities for predicting DIKI. We hypothesize that off-target cellular interactions significantly contribute to DIKI severity [26]. To explore this, we developed a computational framework that combines computed chemical descriptors with predicted off-target interactions. Using an automated AI/ML pipeline, we evaluated multiple ML algorithms. The results demonstrate that integrating physiochemical descriptors with on- and off-target interactions substantially improves the prediction accuracy, including likely severity, and provides insights into the underlying mechanisms of DIKI.

The results of our study confirm that combining chemical descriptors with on- and off-target biological data significantly enhances DIKI predictive accuracy over models that rely solely on one or the other characteristic. This integrative approach offers deeper insights into the biological and chemical interactions associated with DIKI, supporting more informed compound selection and aiding structure−activity relationship (SAR) exploration. Additionally, this model does not rely on clinical exposure parameters such as dose or predicted or identified therapeutic Cmax or AUC values, making it applicable in the early drug discovery process, including the virtual screening phase.

## 2. Results

### 2.1. Statistical Evaluation of Physicochemical Properties in Nephrotoxic (M-DIKI) vs. Non-Nephrotoxic (N-DIKI) Drugs

In this study, we calculated 55 physicochemical properties for a total of 129 M-DIKI (nephrotoxic) drugs and 231 N-DIKI (non-nephrotoxic) drugs (see Supplemental Table S1). The *p*-values from the Mann–Whitney test for nine key physicochemical properties are summarized in Table 1, highlighting statistically significant differences in selected properties (*p* < 0.05). Of these, five properties showed significant differences between M-DIKI and N-DIKI drugs: the apparent permeability in Madin–Darby canine kidney (MDCK) cells, apparent permeability in Caco-2 (human intestinal carcinoma) cells, polar surface area (PSA), fraction of sp^3^-hybridized carbon atoms, and LogD (distribution coefficient). These properties are likely crucial in distinguishing M-DIKI drugs from N-DIKI ones (Table 1).

### 2.2. Correlation Analysis of Physicochemical Properties in DIKI Prediction

**Figure 1:**
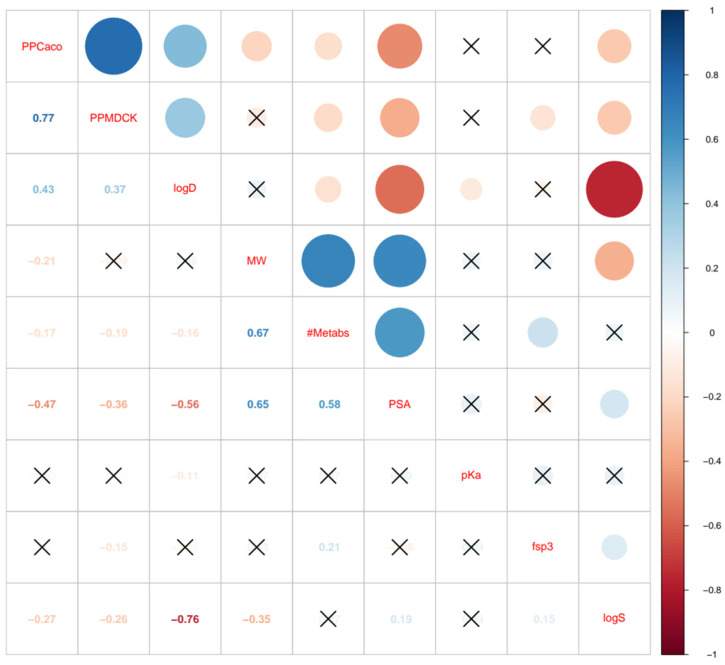
Correlation plot showing relationships between selected molecular properties during feature selection.

Figure 1 presents a correlation plot visualizing the relationships between various molecular properties selected during feature selection. Each cell in the plot represents the correlation coefficient between two properties, with the size and color of the circles indicating the strength and direction of these correlations. Positive correlations are represented in blue, with larger circles indicating stronger relationships. For example, cell permeability in Caco-2 cells and MDCK cell permeability show a strong positive correlation (0.77), suggesting that an increase in one feature correlates with increased relevance in the other features. In contrast, negative feature correlations are depicted in red, with larger circles, signifying stronger negative correlations, such as the strong negative correlation between logS and logD (−0.76), which indicates increased lipophilicity (logD); thus, the solubility tends to decrease. Insignificant correlations of features are marked with an “X”, indicating little to no linear relationship between those pairs of molecular properties.

This analysis highlights that not all selected physicochemical properties independently distinguish between M-DIKI and N-DIKI compounds. This observation underscores the importance of a feature selection process that accounts for intercorrelations between descriptors and selects the most predictive combination of features.

For example, although properties such as Caco-2 and MDCK cell permeability are strongly correlated, they are not selected together in the context of other properties like MW and *fsp^3^*. This approach helps to prevent model bias due to redundant information. Similarly, while log S and log D are significantly correlated, they differ in their relationships with other properties. For instance, log D is not correlated with MW but has a negative correlation with log S. Additionally, *fsp^3^* correlates with log D but not with log S.

Taken together, by carefully selecting descriptors for our model, we can enhance the predictive power by focusing on a diverse compound training set which supports more effective distinguishment between M-DIKI and N-DIKI compounds. However, for some M-DIKI and N-DIKI compounds, statistically significant properties (such as *fsp^3^*, logD, PSA, Caco-2, and MDCK cells permeability) were observed both above and below their mean values. Therefore, we investigated whether off-target interactions, either alone or in combination with these physicochemical properties, could provide a clearer distinction between M-DIKI and N-DIKI drugs.

### 2.3. Predicted Target Interactions for M-DIKI and N-DIKI Drugs

We predicted a total of 6064 interactions for 360 drugs involving 1294 human proteins, with an average of 16.8 protein interactions per drug (see Supplemental Table S2). Among these, 3588 interactions (59%) were confirmed in vitro, while the remaining 2476 (41%) were either unconfirmed or not reported in the literature. Specifically, 1743 interactions were associated with the 129 M-DIKI drugs, averaging 13 interactions per drug and involving 746 proteins. Of these interactions, 939 (53%) were confirmed in vitro, while the remaining 804 (47%) were either unreported or unassessed. In comparison, 4321 interactions were linked to the 231 N-DIKI drugs, averaging 18 interactions per drug and involving 1048 protein targets. Among these, 2649 (61%) interactions were confirmed in vitro, with 1672 (39%) remaining unreported or unassessed.

**Figure 2:**
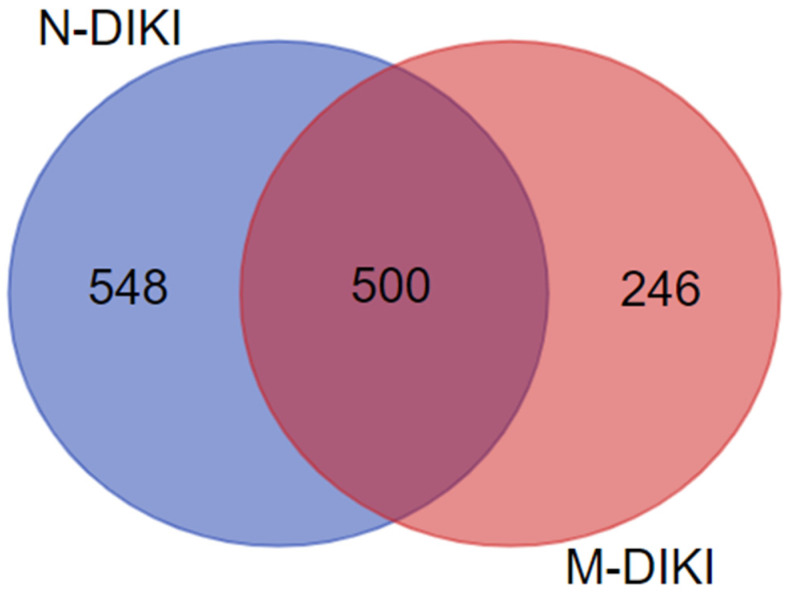
Venn diagram showing the overlap in protein interactions between N-DIKI and M-DIKI compounds. The blue circle represents 548 unique proteins associated with N-DIKI compounds, while the red circle represents 246 unique proteins associated with M-DIKI compounds. The overlap between the two circles indicates 500 proteins shared by both N-DIKI and M-DIKI compounds.

Of the 1299 unique proteins identified, 501 interacted with both nephrotoxic and non-nephrotoxic drugs, suggesting that these interactions may not be useful for distinguishing between M-DIKI and N-DIKI drugs. However, 248 protein targets were unique to M-DIKI drugs, while 550 were unique to N-DIKI drugs (Figure 2). Manually analyzing five physicochemical properties and off-target interactions for a large number of compounds is impractical. Therefore, we applied ML methods to develop predictive models that integrate these chemical and biological interactions, enabling more efficient and accurate predictions across a broad range of discovery compounds.

### 2.4. Performance of AI/ML-Based Drug-Induced Kidney Injury (DIKI) Prediction Models

In our AI/ML-based DIKI prediction framework, we evaluated the performance of various models using combinations of physicochemical properties and off-target interactions. Table 2, Table 3 and Table 4 compare the performance metrics of models that utilized off-targets combined with physicochemical properties (Table 2), off-targets alone (Table 3), and physicochemical properties alone (Table 4). Each table presents results from four ML models: RF, NN, SVM, and RLR.

The models were assessed using several key metrics: sensitivity (recall), specificity, positive predictive value (PPV or precision), negative predictive value (NPV), balanced accuracy, overall accuracy, positive likelihood ratio (LR+), and area under the ROC curve (AUC). These metrics provide a comprehensive view of the models’ effectiveness and reliability in predicting DIKI. The performance of the models was as follows:

1. Model 1: combined physicochemical properties and off-targets.

When both physicochemical properties and off-target interactions were utilized, the model demonstrated robust performance across all metrics. The sensitivity ranged from 0.77 to 0.83, with the RF method achieving the highest value. The specificity was consistently strong, ranging from 0.72 to 0.8, indicating a good balance between correctly identifying M-DIKI and N-DIKI compounds. The positive predictive value (PPV) and negative predictive value (NPV) were also high, reflecting the model’s reliability in making accurate predictions. The balanced accuracy and AUC values were particularly strong, with combination models (e.g., RLR, NN, SVM) achieving an AUC as high as 0.87, indicating excellent discriminative ability.

2. Model 2: off-targets only.

Using only off-target interactions, the model demonstrated variable performance. The sensitivity was especially high, with the Random Forest (RF) method achieving an impressive 0.97. However, this came at the cost of specificity, which was much lower for RF (0.28), indicating a higher rate of false positives. This trade-off suggests that while off-target interactions are useful for identifying potential nephrotoxicants, they may lead to overprediction without additional context. The low positive predictive value (PPV) further highlights the sensitivity-specificity trade-off. Overall, the model showed moderate balanced accuracy, indicating that off-target data alone may not fully capture the complexity of nephrotoxicity.

3. Model 3: physicochemical properties only.

When using only physicochemical properties, the model’s performance was less effective compared to the others. The sensitivity ranged from 0.59 to 0.72, indicating a reduced ability to identify nephrotoxic compounds. The specificity was moderate, but the exclusion of off-target interactions led to a noticeable drop in performance compared to Model 1. The balanced accuracy and AUC values were also lower, suggesting that physicochemical properties alone may not provide sufficient discriminatory power for nephrotoxicity predictions.

### 2.5. Comparative Insights

The incorporation of off-target interactions, whether used independently or in combination with physicochemical properties, substantially enhanced model performance, particularly regarding the sensitivity, positive predictive value (PPV), and likelihood ratio (LR+). These metrics are crucial for early-stage drug safety evaluations. Table 5 summarizes the performance of the best performing models (RLR + NN + SVM) utilizing combined physicochemical and off-target descriptors (Model 1), off-target interactions alone (Model 2), and physicochemical properties alone (Model 3). The model that integrated both off-target interactions and physicochemical descriptors (Model 1) achieved the best overall results, with an accuracy of 79%, an AUC of 0.87, and an LR+ of 3.66, reflecting a balanced sensitivity of 0.79 and a specificity of 0.78. Conversely, the off-target-only model (Model 2) displayed the highest sensitivity (0.87) and NPV (0.89), but its specificity was lower (0.65), resulting in an LR+ of 2.41. In comparison, the model relying solely on physicochemical properties (Model 3) showed the weakest performance, with an LR+ of 1.93, an AUC of 0.69, and an overall lower accuracy, thereby emphasizing the critical role of off-target interactions in enhancing predictive performance.

The trade-offs observed between the sensitivity and specificity across different models underscore the importance of a balanced approach to DIKI prediction. Including off-target interactions helps mitigate the limitations of relying solely on physicochemical properties, offering a more comprehensive and reliable model.

Overall, these results underscore the value of a multifaceted approach that leverages both physicochemical and off-target data to improve the predictive accuracy and reliability of nephrotoxicity assessments in early drug development.

### 2.6. Comparison of Individual vs. Combination Models

We assessed the performance of individual models (single methods) and combination models (integrating multiple methods) across three distinct prediction frameworks:

- Model 1: physicochemical properties + off-targets;
- Model 2: off-targets only;
- Model 3: physicochemical properties only.

### 2.7. Individual Model Performance

Below is a detailed comparison of the performance of the individual models:

1. Random Forest (RF):
  ○ Model 1: achieved a high sensitivity (0.83) but a slightly lower specificity (0.72), showing a good ability to identify nephrotoxicants, albeit with some false positives.
  ○ Model 2: demonstrated an extremely high sensitivity (0.97) but at the cost of a very low specificity (0.28), indicating overfitting to off-target interactions and resulting in many false positives.
  ○ Model 3: performance was moderate, with a balanced sensitivity and specificity around 0.61 (sensitivity 0.69 and low specificity 0.49), illustrating limitations when relying solely on physicochemical properties.
2. Neural Networks (NNs):
  ○ Model 1: delivered a balanced performance with a sensitivity of 0.79 and specificity of 0.74, making it a reliable method for nephrotoxicity prediction.
  ○ Model 2: maintained strong sensitivity (0.87) with moderate specificity (0.65), performing well with only off-target data.
  ○ Model 3: the performance decreased, with a sensitivity of 0.64 and specificity of 0.48, highlighting the constraints of using physicochemical properties alone.
3. Support Vector Machines (SVMs):
  ○ Model 1: showed balanced accuracy, with a sensitivity of 0.82 and specificity of 0.72, comparable to NNs.
  ○ Model 2: achieved high sensitivity (0.87) but lower specificity (0.62), indicating a slight tendency toward overfitting.
  ○ Model 3: lower performance was noted, with a sensitivity of 0.69 and specificity of 0.47, reflecting the limitations of physicochemical properties alone.
4. Regularized Logistic Regression (RLR):
  ○ Model 1: displayed balanced performance with a sensitivity of 0.77 and specificity of 0.78, similar to other individual models.
  ○ Model 2: exhibited a high sensitivity (0.87) but lower specificity (0.65), again suggesting over-reliance on off-target data.
  ○ Model 3: showed relatively poor performance, with a sensitivity of 0.59 and specificity of 0.65, reinforcing the importance of combining multiple data types.

### 2.8. Combination Model Performance

Below is a comparison of the performance of the combination models:

1. Model 1 (physicochemical properties + off-targets):
  ○ The combination models outperformed individual models, with the balanced accuracy, LR+, and AUC values being notably higher. For instance, the RLR, NN, and SVM combination achieved a balanced accuracy of 0.79, an LR+ of 3.66, and an AUC of 0.87, indicating superior predictive power.
  ○ The inclusion of multiple methods provided robustness against overfitting, resulting in improved positive predictive value (PPV) and NPV compared to individual models.
2. Model 2 (off-targets only):
  ○ While combination models performed well, there was more variability in the specificity. For example, the RLR, NN, and SVM combination achieved a balanced accuracy of 0.74 and an AUC of 0.84, but the specificity (and hence LR+) remained relatively low, suggesting that off-target data alone may not provide sufficient discriminative power.
  ○ The combination approach helped mitigate the overfitting observed in individual models like RF, where the sensitivity was very high, but the specificity suffered.
3. Model 3 (physicochemical properties only):
  ○ Combination models offered some improvement over individual models, with the sensitivity and specificity both reaching up to 0.67 and balanced accuracy and AUC values ranging from 0.66 to 0.73. However, the overall performance remained lower compared to Models 1 and 2, indicating the limited effectiveness of using physicochemical properties alone.

Collectively, the combination models consistently outperformed the individual models across all prediction frameworks, particularly in Model 1, where both physicochemical properties and off-target interactions were utilized. This suggests that a multifaceted approach is essential for enhancing prediction accuracy. While off-target interactions added significant value, their use in isolation introduced a risk of overfitting, which led to a high sensitivity but a reduced specificity in individual models, as seen in Model 2. Overfitting in these cases may result in models that are less generalizable and more prone to false positives. Physicochemical properties alone were insufficient for accurate nephrotoxicity predictions, with both individual and combination models in Model 3 showing lower performance metrics compared to those incorporating off-target data.

These comparisons emphasize the importance of integrating diverse predictive methods to achieve reliable and accurate drug safety predictions.

### 2.9. Volcano Plot Analysis of Gene Associations with Nephrotoxicity Risk

**Figure 3:**
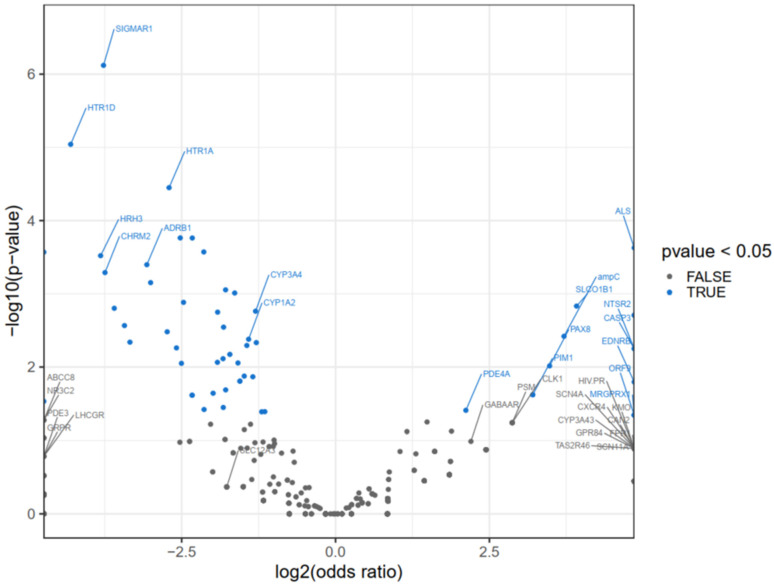
Volcano plot displaying the association between off-targets and their corresponding odds ratios and *p*-values. The *x*-axis represents the log_2_(odds ratio), indicating the strength and direction of the association, while the *y*-axis shows the −log_10_(*p*-value), representing the statistical significance. Blue points represent off-targets with statistically significant associations (*p* < 0.05), while gray points represent non-significant targets. Key off-target genes, such as SIGMAR1, CYP3A4, and HTR1A, show strong associations with higher significance and odds ratios, as labeled.

The volcano plot (Figure 3) illustrates the correlation between specific genes and the probability of a compound being M-DIKI, determined by calculated odds ratios and corresponding *p*-values. The *X*-axis represents the log-transformed odds ratio for each gene. A positive log_2_(odds ratio) (greater than 0) indicates that interaction with the gene increases the likelihood of M-DIKI, whereas a negative log_2_(odds ratio) (less than 0) suggests a protective effect. The *Y*-axis reflects the statistical significance of the odds ratio for each gene, with higher values indicating greater significance. Genes with *p*-values less than 0.05 are highlighted in blue, representing statistically significant associations. Notably, genes such as PDE4A, PIM1, PAX8, SLCO1B1, CASP3, and SIGMAR1, among others, annotated in blue, showed statistically significant associations (*p* < 0.05) with high odds ratios, indicating a strong correlation with inducible nephrotoxicity. Conversely, genes shown in gray are not statistically significant, suggesting that their association with nephrotoxicity may be incidental. Nevertheless, when combined with other off-targets and physicochemical properties, some of these gray-annotated genes demonstrated added predictive value.

### 2.10. Performance Evaluation of DIKI Models Using ROC and Cumulative Gain Analysis

**Figure 4:**
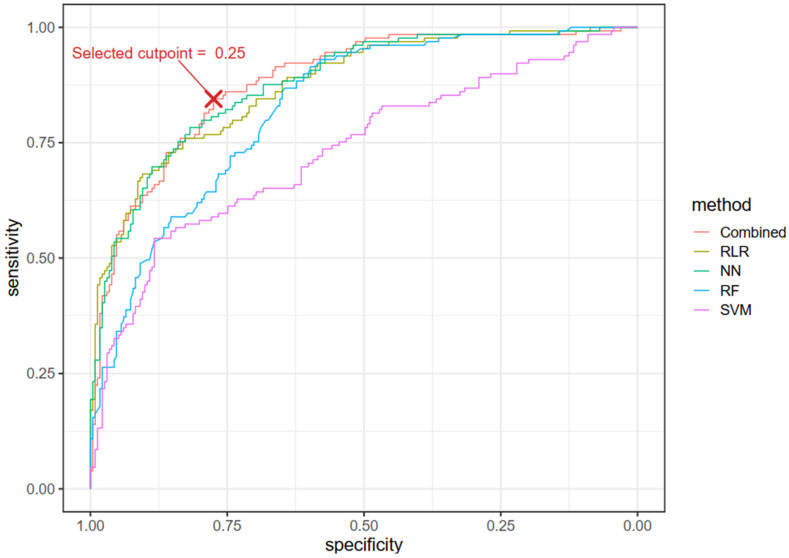
The Receiver Operating Characteristic (ROC) curve demonstrates the balance between sensitivity and specificity for both the combined model (RLR + NN + SVM) and each individual method. The results are based on an instance of 5-fold cross-validation. The red cross indicates the optimal cut-point of 0.25 for the combined model, identified as the point closest to the top-left corner of the ROC curve. At this cut-point for this instance of 5-fold CV, sensitivity is 0.84 and specificity is 0.77. This threshold, established through 5-fold cross-validation, is used to classify compounds as M-DIKI if the score is ≥0.25 and as N-DIKI if the score is <0.25.

**Figure 5:**
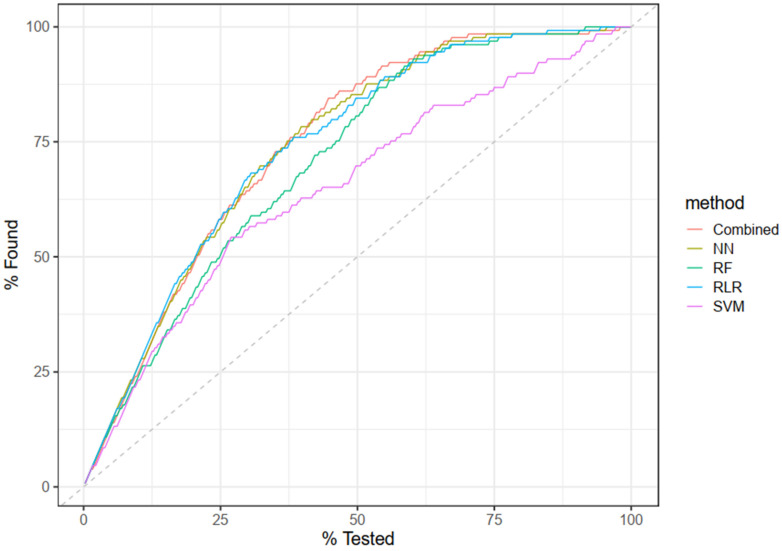
Cumulative gain curve based on an instance of 5-fold CV. This graph illustrates the effectiveness of the proposed model as well as all individual methods in identifying “nephrotoxic” drugs (versus non-nephrotoxic). The *x*-axis represents the cumulative percentage of the total population tested (ranked by the model’s predicted probability for the target class, “nephrotoxic”), while the *y*-axis shows the cumulative percentage of actual “nephrotoxic” cases found up to that point. The curves in the plot indicate the performance of different methods in capturing “nephrotoxic” cases, with higher curves suggesting that the model ranks positive cases (nephrotoxic) effectively toward the top. The dashed diagonal line represents the baseline for random selection, where X% of the population would yield X% of the positive cases.

Based on 100 instances of 5-fold cross-validation for M-DIKI and N-DIKI compounds using the combined model incorporating RLR, NN, and SVM methods, we identified an optimal cut-point of 0.25, corresponding to the closest point to the top-left of the ROC curve (Figure 4). We also conducted a cumulative gain analysis to evaluate the performance of both individual and combined ML models. The gain plot (Figure 5) compares the performance of the combined model with that of the four individual prediction methods, using a single instance of 5-fold cross-validation, clearly demonstrating the superior performance of the combined model compared to the individual models.

## 3. Discussion

The kidneys have a critical role in drug disposition, metabolism, and excretion [27]; therefore, it is expected for DIKI to be a significant attrition factor in preclinical and clinical drug development [28], particularly during the nonclinical and early clinical stages [1,20,29]. Reports from the Innovation and Quality (IQ) Consortium [30] and the New Clinical Development Success Rates highlight alarmingly high failure rates, with 80% of drug candidates failing in discovery and 94% in clinical trials. Kidney toxicity was once reported to account for 2% of drug attrition during nonclinical studies and 9% during clinical development studies [31]. These failures are frequently attributed to adverse interactions with unintended or off-target proteins, as well as pharmacological responses driven by non-validated targets, metabolite interactions, and inadequate physicochemical properties of compounds [32,33]. Despite the availability of screening assays, such as CEREP and kinase profiling, off-target toxicities persist, often due to unknown mechanisms that are difficult to predict or mitigate [30].

The attrition rates from Phase I trials to market approval exceed 90%, especially in therapeutic areas such as oncology, neurology, and endocrinology [34,35]. This underscores the urgent need for advanced strategies to reduce drug development failures. The overall success rate in drug development is approximately 7.9%, with particularly low rates (below 6%) observed in oncology, cardiovascular, and urology. Notably, 30–40% of oncology drugs exhibit renal toxicity during clinical evaluations, in contrast to therapeutic areas like metabolic disorders and infectious diseases, which demonstrate higher success rates [36,37,38]. Nevertheless, kidney toxicity remains a significant concern across all therapeutic areas [39,40].

Approximately 20–30% of drug attrition during early development stages can be linked to nephrotoxicity, with kidney-related adverse drug reactions accounting for 10–15% of reports in clinical trials [19,29]. Given these alarming statistics, integrating computational models early in drug development provides opportunities for predicting and mitigating off-target risks [33,41], increasing the likelihood of successfully advancing promising compounds through the drug development pipeline.

### 3.1. Predicting DIKI with Integrated Systems Toxicology and ML Approaches

To address DIKI, various research groups have employed in vitro [42], in silico, organ-on-a-chip [43], and in vivo [44,45] approaches to develop predictive models. For instance, Kato et al. [44] assessed the use of adult zebrafish as an in vivo model for detecting DIKI. In their study, zebrafish were exposed to 28 nephrotoxicants and 14 non-nephrotoxicants over four days, revealing that 16 nephrotoxicants induced kidney injury. The study reported a sensitivity of 57%, specificity of 100%, positive predictive value of 100%, and negative predictive value of 54%. Additionally, they identified three candidate genes (egr1, atf3, and fos) with increased expression levels associated with kidney toxicity. These findings suggest that adult zebrafish exhibit a nephrotoxic response similar to mammals, making them a feasible model for predictive studies in DIKI.

The EGR genes, particularly EGR1, play a crucial role in regulating various biological processes, including apoptosis and inflammation, especially in the context of nephrotoxicity [46,47,48]. EGR1 influences CASP3 expression, which affects cell survival in the presence of nephrotoxic agents. Furthermore, EGR1’s regulation of PDE4A, CXCR4, CYP1A2, CYP3A4, F3, NR3C2, and SCN11A links inflammatory and metabolic pathways to renal injury.

On the computational front, several in silico models have been developed to predict nephrotoxicity [49,50,51]. The key physicochemical properties contributing to increased promiscuity include lipophilicity (clogP), high molecular weight (MW), the presence of ionizable amines (leading to high pKa), and polar surface area (PSA).

For example, Irvine et al. reviewed in vitro models for DIKI prediction. Their analysis showed a primary focus on the proximal tubule (84%) with minimal attention to the glomerulus/Bowman’s capsule (7%). Our DIKI de-risking models use a generalized approach that addresses both tissues, incorporating chemical and biological interactions. Paine et al. [52] applied ML with PLS and RF models to predict renal clearance, achieving a predictive accuracy above 0.8 based on chemical descriptors. Our model aligns with this, identifying logP and pKa as key features for distinguishing M-DIKI from N-DIKI. Gong et al. developed an SVM model for nephrotoxicity in Chinese herbal and small-molecule drugs, though it was limited by the lack of biological descriptors. Shi et al. [53] created a DIKI prediction model with 86.24% accuracy, identifying molecular weight, PSA, and other properties as nephrotoxicity predictors. Our model agrees, emphasizing LogP, LogS, and PSA while adding biological interaction descriptors for enhanced robustness. Connor et al. compiled the DIRIL database, a comprehensive DIKI dataset. This dataset integrates data from Shi et al. and Gong et al. [54] with duplicates and inorganic compounds removed to retain only drug-like molecules suitable for cheminformatics. Our data align fully with DIRIL (see Supplemental Table S1), as our focus was also on drug-like molecules. Connor et al. [21] found correlations between nephrotoxicity, molecular weight, and lipophilicity but not with daily dose. While our model does not account for daily dose, it identified lipophilicity as the main distinguishing feature between M-DILI and N-DILI, in agreement with the published results.

Lee et al. [55] employed Quantitative Structure–Activity Relationship (QSAR) models to assess tubular necrosis, interstitial nephritis, and tubulo-interstitial nephritis. Their Support Vector Machine (SVM) models achieved over 83% predictive accuracy, highlighting the importance of incorporating metabolite information. In alignment with these findings, our research identified the predicted number of metabolites as a key differentiating descriptor, integrating chemical fingerprints with biological interactions to enhance DIKI predictions.

Additionally, the application of ML in clinical settings shows promise for improving outcomes in AKI and contrast-induced nephropathy (CIN). Recent studies demonstrate the efficacy of Gradient Boosting Machine (GBM) and Support Vector Machine (SVM) models, achieving high area under the curve (AUC) values of 0.87 and 0.85, respectively. This aligns with our exploration of ML applications in DIKI, where the inclusion of key clinical variables enhances model performance. However, the variability observed in models like Random Forest (RF) and XGBoost emphasizes the need for further validation, reflecting the complexities involved in predicting nephrotoxicity.

Moreover, various in vitro models, such as 3D organ-on-chip systems [42,43] and transcriptomics assessments [56,57], have been developed to predict DIKI. While these in vivo, in silico, and in vitro approaches provide valuable insights for early risk mitigation, they often lack integration into a comprehensive systems toxicology approach. Additionally, in contrast to the work described here, current published models have not sufficiently considered off-target interactions in different kidney compartments to fully contextualize DIKI.

Therefore, we propose that integrating all these approaches into a single framework—a systems toxicology approach—will enhance predictive accuracy and reduce attrition due to DIKI. Specifically, our in silico integration study demonstrates the enhanced performance of AI/ML models that combine both physicochemical properties (chemistry) and predicted off-target interactions (biology/pharmacology) for DIKI prediction. The high AUC value of 0.87 highlights the significant improvement in predictive accuracy when these features are integrated. This finding underscores the importance of leveraging both physicochemical data and off-target interactions to better predict DIKI. Future analyses should compare these integrated models with existing approaches to provide a clearer evaluation of their advantages.

### 3.2. Biological Relevance of Target Interactions

Our analysis identifies key off-target interactions (as shown in the volcano plot) and specific physicochemical properties that play critical roles in predicting DIKI. Recognizing these interactions provides valuable insights for optimizing drug design and enhancing DIKI safety profiles. Compared to the existing literature, our findings offer new perspectives on how certain off-target interactions contribute to DIKI. Below, we discuss a few predicted targets and their relevance to DIKI:

CASP-3: Lan et al. [58] investigated the role of caspase-3 in ischemia–reperfusion injury (IRI), a significant risk factor for chronic renal failure. Their study revealed that caspase-3, a key driver of apoptosis, contributes to early microvascular damage in AKI. In a mouse model, caspase-3 knockout (caspase-3-/-) mice demonstrated improved long-term kidney outcomes, including reduced fibrosis and preserved renal function, despite more severe initial tubular injury. These findings underscore caspase-3’s activator role in post-IRI microvascular dysfunction and align with our AI/ML model, which identified caspase-3 as a key biological descriptor and potential predictor of DIKI.
SLC12A3: Our analysis also identified SLC12A3, a gene encoding the sodium–chloride (Na-Cl) symporter, as a key target in predicting DIKI. SLC12A3 plays a critical role in renal electrolyte balance, and its inhibition is associated with adverse renal events, as demonstrated in both clinical trials and case reports.

In a Phase II study of chlorthalidone, a Na-Cl symporter inhibitor, 160 patients with hypertension and advanced chronic kidney disease (CKD) were evaluated. The study revealed that AKI occurred in 41% of patients receiving chlorthalidone compared to only 13% in the placebo group. Other common adverse events (AEs) included asymptomatic orthostatic hypotension, dizziness, and hypomagnesemia. This suggests that inhibiting SLC12A3 may contribute to increased renal vulnerability, particularly in CKD patients [59]. Additionally, a case report highlighted the severe electrolyte disturbances that can result from Na-Cl symporter inhibitors. A 52-year-old man who was prescribed indapamide [60], another Na-Cl symporter inhibitor, developed hypokalemia, hypocalcemia, hyponatremia, and other imbalances, leading to seizures and AKI. This case further emphasizes the renal and systemic risks associated with Na-Cl symporter inhibition.

Moreover, the UK Medicines and Healthcare products Regulatory Agency (MHRA) outlines specific safety concerns for chlortalidone, including azotemia, hyponatremia, hypokalemia, and various electrolyte disturbances, all of which are significant contributors to kidney injury. These effects reinforce the need for caution when using Na-Cl symporter inhibitors in patients at risk of renal impairment.

Taken together, these findings suggest that SLC12A3 inhibition may predispose patients to DIKI through electrolyte imbalances and kidney stress, aligning with our model’s predictions, highlighting its importance in differentiating DIKI vs. non-DIKI.

### 3.3. Endothelin Receptors (ETA/ETB)

Our analysis identified endothelin receptors ETA and ETB as key targets linked to DIKI. Endothelin receptor antagonists (ERAs), such as macitentan, are used to treat cardiovascular and pulmonary conditions, but their renal effects raise concerns.

In a Phase II study of macitentan in 91 patients with heart failure and pulmonary vascular disease, 5.49% experienced AKI (NCT03714815), alongside other serious adverse events like pneumonia and atrial fibrillation. Another study in patients with chronic thromboembolic pulmonary hypertension reported kidney-related AEs, including serious hematuria.

Preclinical studies in polycystic kidney disease (PKD) models further confirmed the risks, showing that blocking ETB receptors accelerated renal fibrosis and impaired kidney function. These findings align with our AI/ML model, which identified endothelin receptors as predictors of DIKI.

### 3.4. ML-Based DIKI Predictions for Drugs with Varied Mechanisms of Action

The violin plots in Figure 3 display the predicted probabilities of DIKI for eight FDA-approved or withdrawn compounds: Doxorubicin (a DNA-topoisomerase inhibitor, approved), Carfilzomib (a 20S proteasome inhibitor, approved) [61], Gentamicin (a 30S ribosomal protein inhibitor, approved), Methotrexate (a dihydrofolate reductase inhibitor, approved), Troglitazone (a PPARγ agonist, withdrawn), Metoprolol (a beta-adrenoceptor antagonist, withdrawn), Dopamine (a Dopamine receptor agonist, registered), and Sarcolysin (a DNA-alkylating agent, approved). These compounds were selected to represent diverse biological mechanisms, each associated with either known M-DIKI or N-DIKI classification. The predictions were generated using Model 1, which incorporates off-target interactions and physicochemical properties to assess DIKI risk. Each violin plot corresponds to one compound, showing the distribution of DIKI probability scores across 100 replicates of 5-fold cross-validation. The dots within each plot represent individual replicates. Methotrexate and Carfilzomib show distinct probability patterns. Methotrexate consistently shows lower nephrotoxicity probabilities (~0.25), while Carfilzomib shows higher probabilities (>0.8) in most replicates. Despite these differences, the model classifies both compounds as M-DIKI based on our score cutoff of 0.25, aligning with the FDA’s classification. In contrast, Gentamicin and Doxorubicin display consistently high nephrotoxicity probabilities (>0.9) across all 100 replicates, indicating a stronger nephrotoxic potential compared to Methotrexate and Carfilzomib. This also matches the FDA’s classification of these compounds as M-DIKI. Troglitazone, Metoprolol, Dopamine, and Sarcolysin, on the other hand, were consistently predicted as N-DIKI, with probability scores well below 0.25 across all replicates, again in agreement with their FDA classification as N-DIKI.

**Figure 6:**
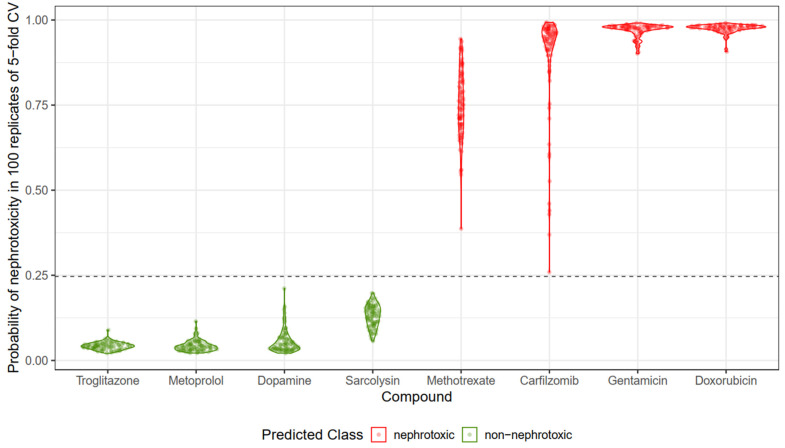
Violin plots showing the predicted probabilities of Drug-Induced Kidney Injury (DIKI) for eight FDA-approved or withdrawn compounds. The distribution of DIKI probability scores is based on 100 replicates of 5-fold cross-validation. Compounds classified as nephrotoxic (M-DIKI) have probabilities above the cutoff of 0.25 (in red), while non-nephrotoxic (N-DIKI) compounds show probabilities below this threshold (in green).

We identified 17 interactions for Carfilzomib, including PSMB5, 26S, ABCC3, ABCC2, PSM, PSMB8, PSMB9, HIV.PR, REN, PSMB, CTSD, 20SIP, PSMB10, PSMB2, PSMB1, PSMB6, and PSMB7 (see Supplemental Table S2). Both primary therapeutic targets and 15 off-target DIKI interactions were accurately predicted. The shared pathogenesis of Carfilzomib’s nephrotoxicity and cardiovascular toxicity may involve the renal endothelium, as supported by studies of isolated endothelial cells and human renal biopsies. Notable predicted DIKI interaction targets include CTSD (Cathepsin D), ABCC3 (ATP-binding cassette subfamily C member 3), and REN (renin), all expressed in the renal endothelium and known non-selective pharmacologic targets. The predicted score is >0.8, indicating DIKI potential (See Figure 6).

KZR-616 (Zetomipzomib) is a highly selective, irreversible inhibitor of LMP7 and LMP2 [62], representing a new class of proteasome inhibitors. Its nonclinical toxicity profile was not disclosed at the time of this manuscript. KZR-616 has been tested in patients with autoimmune hepatitis and lupus nephritis (LN). The PALIZADE trial, a global placebo-controlled Phase 2b study, aimed to evaluate the efficacy and safety of KZR-616 in active LN but was placed on hold by the sponsor and the FDA due to four Grade 5 serious adverse events (SAEs) in one placebo patient and three patients in the low- or high-dose experimental group [63]. We conducted in silico predictions of DIKI interactions for KZR-616 and identified nine interactions (PSMB8, PSM, 20SIP, ABCC2, ABCC3, GHSR, PSMB5, PSMB9, PSMB2, and PSMB7). The primary pharmacologic targets, LMP7/PSMB8 and LMP2/PSMB9, were included in this analysis. However, all off-target interactions fell outside the 38 descriptors relevant to M-DIKI, suggesting a predicted score of less than 0.2, indicating that KZR-616 may not present a significant DIKI risk in nonclinical models and/or healthy human volunteers. Currently, no conclusive clinical studies link KZR-616 to potential human DIKI risk.

To further improve the model’s accuracy in distinguishing between M-DIKI and N-DIKI for novel compounds, additional validation with a broader set of well-characterized DIKI compounds is recommended. As part of ongoing model refinement, we regularly incorporate new ML results from confirmed DIKI predictions for newer compounds into the training data. This continuous feedback loop is essential for enhancing the reliability and utility of the model’s DIKI predictions.

### 3.5. Limitations of the DIKI Predictive Model

While our DIKI predictive model achieves approximately 80% balanced accuracy, several limitations hinder its effectiveness. The challenges include dataset quality, off-target predictions, validation processes, data integration, and DIKI projections.

### 3.6. Dataset Limitations

The quality of our dataset is crucial; however, biases in source data and a limited number of defined DIKI outcomes (only 360 molecules) reduce the prediction accuracy for novel compounds. Small or non-diverse datasets can lead to overfitting, further limiting practical application.

### 3.7. Challenges in Off-Target Predictions

Off-target prediction methods, such as the Off-Target Safety Analysis (OTSA), are constrained by the targets they include. Many targets remain unidentified, and predictions are heavily dependent on the availability of in vitro and in vivo data. Compounds like cisplatin complicate modeling efforts due to the scarcity of force-field parameters and SAR data, which limits predictive power. High-molecular-weight molecules, such as amphotericin B, introduce additional challenges, as off-target prediction systems often lack sufficient SAR data to accurately identify their structural neighbors, making off-target predictions more difficult. Additionally, since pharmaceutical companies typically do not disclose off-target interactions until a drug is approved, the predicted off-target interactions may be accurate but remain unverifiable. We recommend continuously updating the model with newly published data as soon as drugs are approved.

### 3.8. Validation and Resource Intensity

To enhance confidence, we employ multiple ML models, but this can be resource-intensive for large datasets. Ambiguous predictions require careful interpretation, and we utilize a weight-of-evidence (WoE) approach to prioritize confident predictions, albeit with necessary assumptions regarding DIKI potential.

### 3.9. Data Integration Challenges

Data integration faces hurdles, including inconsistencies between sources, differing standards, and a lack of contextual information. These issues complicate result interpretation and affect the accuracy of DIKI projections, particularly regarding complex renal injury mechanisms.

### 3.10. Species Differences and Predictive Limitations

Translating animal data to humans is complicated by species differences in kidney responses. Our predictions, based on human in vitro data, lack animal data, limiting our ability to predict interactions for preclinical species. Many ML models still struggle to integrate these complexities, affecting prediction reliability relative to compound-induced pathway-specific outcomes.

### 3.11. Future Directions

To enhance our understanding of off-target interactions, we recommend combining multi-omics data, particularly transcriptomics and proteomics, in future models. Our current focus is on potential direct off-target interactions related to DIKI, but we acknowledge the constraints imposed by the chemistry and SAR of certain targets. To address these limitations, we propose integrating data from orthogonal omics methodologies and leveraging transfer learning to improve model performance, especially in data-scarce scenarios. Currently, our method identifies direct off-targets but does not predict downstream differentially expressed genes (DEGs), a limitation in comparison to methods like Tox-GAN, which can predict gene expression patterns but fail to identify relevant off-target interactions. To enhance our predictions, we propose an integrated framework that combines our predicted off-targets with Tox-GAN-generated DEG signatures [64] and L1000 data [65]. This approach will improve our understanding of off-target binding and the adverse pathways associated with M-DILI compounds. In our next-generation modeling, we propose to create a comprehensive DIKI prediction model that incorporates off-target predictions, L1000 gene signature data [65], proteomics predictions, and predicted gene expression signatures, ultimately improving accuracy and the understanding of kidney toxicity.

These future approaches build upon the results presented here showing that integrating physicochemical properties with on- and off-target interactions significantly improved DIKI prediction accuracy using an AI/ML approach when compared to other predictive models that rely on chemical properties alone.

## 4. Materials and Methods

### 4.1. Data Collection and Preprocessing

The dataset for this study consisted of 360 compounds, with 231 classified as non-nephrotoxic and 129 as nephrotoxic by the FDA database. The data were compiled from multiple sources, incorporating both off-target interaction data and physicochemical properties. This comprehensive dataset included 9 physicochemical descriptors and 1415 gene interaction indicators, providing a rich feature space for model development.

### 4.2. Off-Target Prediction Methods

The target prediction process follows a structured, multi-step approach. The first step employs two-dimensional (2D) chemical similarity analysis, where 360 compounds (231 N-DIKI and 139 M-DIKI) curated by the FDA were assessed for potential off-target interactions [1,26]. This analysis utilizes an array of cheminformatics and ML techniques (Chemotargets-CLARITY, Barcelona), including Similarity Active Subgraphs (SASs) [66], SAS-based Quantitative Structure–Activity Relationship (SAR) models [67], Molecular Similarity (SIM) [68,69], the Similarity Ensemble Approach (SEA) [70,71], ML methods, and Cross-Pharmacology Index (XPI) [72]. Among the ML methods employed [73,74] are Random Forest (RF) [75], Artificial Neural Networks (aNNs) [76], and Support Vector Machines (SVMs) [75]. Together, these tools form the Off-Target Safety Analysis (OTSA) [33], which predicts off-target binding that could either yield therapeutic benefits or result in unintended adverse effects.

Below is a breakdown of the six cheminformatics methodologies used in the target prediction process:

- SAS: This method focuses on identifying the smallest active subgraph that contains the minimum pharmacophoric features required for biological activity. By doing so, it reveals molecular pairs that were once considered dissimilar, expanding their applicability and improving prediction accuracy. SAS also helps reduce false positives by preventing the identification of similar artifacts, thus enhancing precision.
- SAR: SAR enables the development of large-scale QSAR (Quantitative Structure–Activity Relationship) models, particularly useful for specific target families such as kinases, GPCRs, ion channels, proteases, transporters, and immunoglobulin receptors.
- SIM: This methodology computes chemical similarity using three distinct 2D descriptors: Pharmacophoric Fragments (PHRAGs), Feature-Pair Distributions (FPDs), and Shannon Entropy Descriptors (SHEDs). Each descriptor introduces a different level of randomness, creating a balanced view of structural similarity.
- SEA: SEA uncovers related proteins by analyzing the set-wise chemical similarity among their ligands. This approach has proven effective in predicting novel ligand–target interactions, even when relying solely on chemical structure data.
- MLM: ML models (MLMs) utilize over a thousand classifiers based on FPD molecular descriptors for qualitative binding predictions. MLM generates a consensus score from three models (aNN, SVM, and RF). If the score is positive, the ligand–target interaction is deemed likely.
- XPI: XPI harnesses cross-pharmacological data from thousands of small molecules acting on various biological targets. This enables an in-depth, comprehensive analysis of cross-pharmacology.

To determine the similarity of specific compounds to those in our reference database, we employed the Tanimoto similarity distance to compare SAS fingerprints. This metric was also applied in SAR, SIM, XPI, and SEA analyses to identify related compounds within the database.

### 4.3. Feature Selection

To identify the most relevant features for classification, we employed Lasso Logistic Regression [77] using the glmnet package in R. The Lasso method applies L1 regularization, which helps in feature selection by shrinking less important coefficients to zero. To ensure the robustness of the feature selection process, a 5-fold cross-validation procedure was repeated 100 times. This rigorous approach led to the selection of 42 features, including 4 physicochemical descriptors and 38 gene interaction indicators, which were then used as input for subsequent model training and evaluation.

Selected Physicochemical Properties

Physicochemical propertiesTotal number of metabolitesPolar surface area (PSA)Fraction of sp3-hybridized carbon atoms (*fsp3*)pKa

These properties, along with the selected gene interaction indicators, were crucial in improving the predictive performance of the models by focusing on the most informative features.

Selected Genes

PDE4AADRB1GABAARPSMABCC8ORF9CYP3A43CAN2NR3C2HTR1ACYP3A4CASP3ALSGPR84MRGPRX1SLCO1B1HRH3SIGMAR1PDE3CXCR4GRPRNTSR2SCN11ATAS2R46HIV.PRPIM1SCN4ACYP1A2CLK1SLC12A3KMO
CHRM2HTR1DampCPAX8EDNRBLHCGRFPR1


These genes play a key role in the biological processes related to nephrotoxicity and were thus essential in enhancing the model’s ability to classify compounds effectively. It is noteworthy that not all selected genes or physicochemical properties are individually capable of significantly distinguishing between nephrotoxic and non-nephrotoxic compounds. This suggests that the feature selection process considers the intercorrelations among different descriptors and selects those that, when combined, are most predictive.

Below are the definitions of the performance metrics we used to evaluate the different methods:

- Sensitivity (recall): Sensitivity, also known as recall, measures the proportion of actual positive cases that were correctly identified by the model. It indicates the model’s ability to detect true positives.
  $$\mathrm{Sensitivity}=\frac{\mathrm{True}\;\mathrm{Positives}\;\backslash \backslash (\mathrm{TP}\backslash \backslash )}{\mathrm{True}\;\mathrm{Positives}\;\backslash \backslash (\mathrm{TP}\backslash \backslash )+\mathrm{False}\;\mathrm{Negatives}\;\backslash \backslash (\mathrm{FN}\backslash \backslash )}$$
- Specificity: Specificity measures the proportion of actual negative cases that were correctly identified by the model. It reflects the model’s ability to avoid false positives by correctly identifying true negatives.
  $$\mathrm{Specificity}=\frac{\mathrm{True}\;\mathrm{Negatives}\;\backslash \backslash (\mathrm{TN}\backslash \backslash )}{\mathrm{True}\;\mathrm{Negatives}\;\backslash \backslash (\mathrm{TN}\backslash \backslash )+\mathrm{False}\;\mathrm{Positives}\;\backslash \backslash (\mathrm{FP}\backslash \backslash )}$$
- Positive predictive value (PPV or precision): PPV, also known as precision, represents the proportion of positive predictions that were actually correct. It is a measure of the model’s accuracy in predicting positive cases.
  $$\mathrm{PPV}=\frac{\mathrm{True}\;\mathrm{Positives}\;\backslash \backslash (\mathrm{TP}\backslash \backslash )}{\mathrm{True}\;\mathrm{Positives}\;\backslash \backslash (\mathrm{TP}\backslash \backslash )+\mathrm{False}\;\mathrm{Positives}\;\backslash \backslash (\mathrm{FP}\backslash \backslash )}$$
- Negative predictive value (NPV): NPV measures the proportion of negative predictions that were correct. It indicates how well the model predicts negative cases.
  $$\mathrm{NPV}=\frac{\mathrm{True}\;\mathrm{Negatives}\;\backslash \backslash (\mathrm{TN}\backslash \backslash )}{\mathrm{True}\;\mathrm{Negatives}\;\backslash \backslash (\mathrm{TN}\backslash \backslash )+\mathrm{False}\;\mathrm{Negatives}\;\backslash \backslash (\mathrm{FN}\backslash \backslash )}$$
- Balanced accuracy: Balanced accuracy is the average of sensitivity and specificity, making it particularly useful in scenarios where the classes are imbalanced. It provides a more nuanced view of the model’s performance by giving equal weight to both classes.
  $$\mathrm{Balanced}\;\mathrm{Accuracy}=\frac{\mathrm{Sensitivity}+\mathrm{Specificity}}{2}$$
- Accuracy: Accuracy is the overall proportion of correct predictions, including both true positives and true negatives. It provides a general measure of the model’s performance across all classes.
  $$\mathrm{Accuracy}=\frac{\mathrm{True}\;\mathrm{Positives}\;\backslash \backslash (\mathrm{TP}\backslash \backslash )+\mathrm{True}\;\mathrm{Negatives}\;\backslash \backslash (\mathrm{TN}\backslash \backslash )}{\mathrm{Total}\;\mathrm{Population}\;\backslash \backslash (\mathrm{TP}+\mathrm{TN}+\mathrm{FP}+\mathrm{FN}\backslash \backslash )}$$
- Area under the curve (AUC): AUC is a measure of the model’s ability to distinguish between classes. It is the area under the Receiver Operating Characteristic (ROC) curve, which plots the true positive rate (TPR) against the false positive rate (FPR) at various threshold settings. A higher AUC indicates better model performance.

$$\mathrm{AUC}=\int_{0}^{1}\mathrm{TPR}\left(FPR\right)d\left(\mathrm{FPR}\right)$$

where TPR is the true positive rate, and FPR is the false positive rate.

- Positive likelihood ratio (LR+): The ratio of the probability of a positive test result in true positives (nephrotoxic cases) to the probability of a positive test result in false positives (non-nephrotoxic cases). A higher LR+ indicates greater confidence that a positive prediction corresponds to an actual positive case.

These metrics collectively provide a comprehensive understanding of the models’ strengths and weaknesses, helping to identify the most effective approaches for predicting nephrotoxic compounds.

### 4.4. Establishing and Validating a Probability Cutoff to Balance Sensitivity and Specificity

We set the probability cutoff at 0.25 to achieve a balance of approximately 80% specificity while maintaining high sensitivity in our study. This cutoff is flexible and can be adjusted for different toxicological endpoints. In exploratory studies, a lower cutoff like ours can help identify additional nephrotoxic compounds by increasing sensitivity. Conversely, a higher cutoff may be more suitable in preclinical settings, where minimizing the exclusion of promising drug candidates is crucial.

Our extensive cross-validation analysis strengthens the reliability of this cutoff, demonstrating its effectiveness in capturing the predictive capabilities of our ensemble model. Additionally, by integrating various ML approaches, we established the robustness of our predictions, making the model adaptable to diverse needs in early discovery, preclinical research, and clinical applications.

To determine this threshold, we conducted 100 replicates of 5-fold cross-validation and used the median of these thresholds in our analysis.

All analyses were conducted using R (version 4.1.1), a powerful statistical computing environment widely used for data analysis and ML. The following R packages were employed to implement and evaluate the models:

- caret (version 6.0-92): This package was used for training, tuning, and evaluating ML models. It provides a unified interface for model training and performance assessment across different algorithms.
- glmnet (version 4.1-7): Used for implementing Ridge Logistic Regression (RLR), this package handles the regularization process and cross-validation, ensuring the model’s robustness and reducing overfitting.
- randomForest (version 4.7-1.1): This package was employed to implement Random Forest models, facilitating the selection of optimal features and enhancing prediction accuracy through ensemble learning.
- nnet (version 7.3-17): Used for training Neural Networks, this package allowed for the fine-tuning of model parameters such as the number of hidden units and the decay parameter, enabling the model to learn complex patterns within the data.
- e1071 (version 1.7-9): This package was utilized to implement Support Vector Machines (SVMs), including the tuning of cost and kernel parameters to optimize classification performance.
- pROC (version 1.18.0): This package was essential for ROC curve analysis and threshold optimization. It provided tools for assessing the models’ discriminatory power and selecting the optimal decision boundary based on the ROC curve.

The hyperparameters used for machine model development are listed below:

- RF: mrty = 7.
- NN: size = 9; decay = 0.1.
- SVM: sigma = 0.0625; C = 2.
- RLR: lambda = 0.02417371; alpha = 0.

The glmnet model, which combines the three methods (RLR, NN, SVM), uses the following hyperparameters for RLR:

- RLR in the combined model: lambda = 0.00713962; alpha = 0.

These values are included in the R package.

By leveraging these specialized packages, we ensured a rigorous and comprehensive analysis, leading to reliable and interpretable results across the various ML models. Multiple ML models were trained and fine-tuned using a set of carefully selected features. The models employed included Ridge Logistic Regression (RLR), Neural Networks (NNs), Random Forests (RFs), and Support Vector Machines (SVMs). Given the unbalanced nature of the dataset, which contained a higher proportion of non-nephrotoxic compounds compared to nephrotoxic ones, a threshold-shifting approach was adopted to optimize the decision boundary. This approach was necessary to ensure that the models could accurately distinguish between nephrotoxic and non-nephrotoxic compounds despite the imbalance. To determine the optimal cut-point, we used a strategy that minimized the function (1 − “sensitivity”) ^2^ + (1 − “specificity”)^2^. This metric corresponds to the point on the ROC curve that is closest to the top-left corner, representing the best balance between sensitivity and specificity

For each model, hyperparameters and thresholds were carefully tuned using 20 replicates of 5-fold cross-validation to ensure robust and reliable performance:

1. Ridge Logistic Regression (RLR): Implemented using the glmnet package, with the regularization parameter (lambda) fine-tuned through cross-validation. This process helps control the trade-off between model complexity and accuracy, ensuring that the model generalizes well to unseen data [78].
2. Neural Networks (NNs): Trained using the nnet package, with a grid search approach employed to optimize the number of hidden units and the decay parameter. The hidden units determine the model’s capacity to capture complex patterns, while the decay parameter prevents overfitting by adding a penalty to large weights.
3. Random Forest (RF): Implemented using the randomForest package, with the number of variables randomly sampled at each split (mtry) tuned through grid search. This tuning is crucial for balancing the trade-off between bias and variance, enabling the model to make accurate predictions while remaining resilient to noise.
4. Support Vector Machines (SVMs): Implemented using the e1071 package, with the cost (C) and kernel width (sigma) parameters optimized via grid search within the cross-validation framework. The cost parameter controls the trade-off between maximizing the margin and minimizing classification errors, while the kernel width defines the decision boundary’s shape in the feature space.
5. Ensemble Model: To enhance predictive accuracy, eleven ensemble models were created by combining the probabilities (scores) from all possible combinations of the individual models (RLR, NN, RF, and SVM). Ridge Logistic Regression was employed to aggregate these scores, producing a final classification that leverages the strengths of each model.

This comprehensive tuning and ensemble approach ensured that the final models were both accurate and generalizable, capable of making reliable predictions across a variety of datasets. To evaluate both the tuning procedure and the predictive accuracy of the models, the dataset was split into two parts. Approximately 70% of the dataset, comprising 162 non-nephrotoxic and 90 nephrotoxic compounds, was designated as the training set. This set was used for the tuning process. The remaining 30%, consisting of 69 non-nephrotoxic and 39 nephrotoxic compounds, was reserved as an independent test set for final evaluation.

During the tuning process, the models were trained on the training set using various hyperparameters and thresholds identified through 5-fold cross-validation.

For each model, hyperparameters and thresholds were tuned using 20 replicates of 5-fold cross-validation, with the following steps:

- Step 1: randomly divide the dataset into 5 folds of roughly equal sizes, ensuring that each fold maintains the same proportion of nephrotoxic and non-nephrotoxic compounds as in the original dataset to preserve class balance.
- Step 2: select one fold as the validation set while combining the remaining four folds to form the training set.
- Step 3: train the model on the training set, tuning hyperparameters and thresholds based solely on the training data.
- Step 4: evaluate the model on the validation fold, recording metrics such as sensitivity and specificity for that fold.
- Step 5: repeat Steps 2–4 for each of the 5 folds so that each fold serves as the validation set once.
- Step 6: aggregate performance metrics (e.g., average sensitivity and specificity across folds) to assess the model’s stability and generalizability.

This approach ensures comprehensive tuning and evaluation, reducing bias from single validation splits and supporting robust hyperparameter selection.

After the optimal hyperparameters and thresholds were determined, the models were retrained on the entire training set using these best settings. These retrained models were then applied to the independent test set to assess their performance on unseen data.

The performance metrics evaluated included the sensitivity (recall), specificity, negative predictive value (NPV), positive predictive value (PPV or precision), accuracy, area under the ROC curve (AUC), and balanced accuracy. These metrics provided a comprehensive understanding of each model’s ability to correctly classify nephrotoxic and non-nephrotoxic compounds.

To ensure the robustness of our findings, the entire process of splitting the data into training and test sets was repeated 100 times. For each iteration, the performance metrics were computed, and then their medians were computed across all iterations. These median metrics are reported as the final performance measures, providing a reliable estimate of the models’ predictive capabilities.

**Figure 7:**
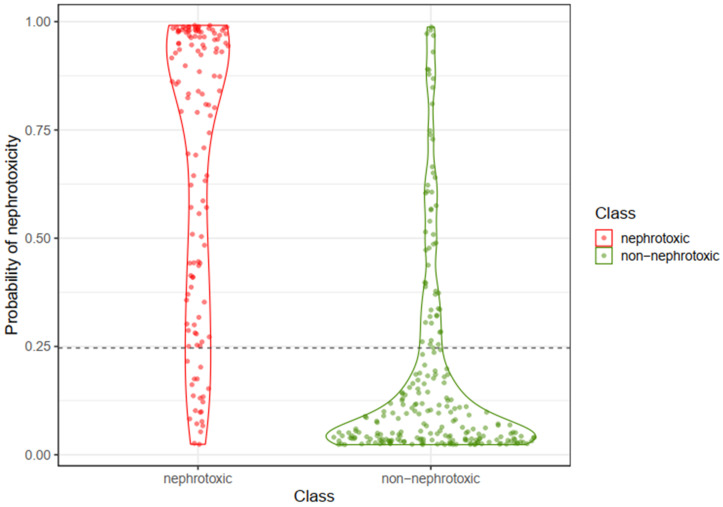
Violin plots showing the probability of nephrotoxicity for nephrotoxic (red) and non-nephrotoxic (green) compounds, based on one instance of 5-fold cross-validation. The dashed horizontal line at a probability score of 0.25 indicates the optimal cut-point for classification. Points within the red region indicate nephrotoxic compounds, while points within the green region indicate non-nephrotoxic compounds.

Figure 7 shows violin plots of the probability of nephrotoxicity (based on one instance of 5-fold cross-validation) for nephrotoxic and non-nephrotoxic compounds using the combined RLR, NN, and SVM methods.

The best model is a combination of the outcomes from three ML approaches (RLR, NN, and SVM) that use both off-targets and physicochemical properties. The formula to compute the probability score for N-DIKI or M-DIKI is as follows:

$$Probability\left(\mathrm{nephrotoxicity}\right)=\frac{1}{1+e^{-\left(-8.0+1.4\times \mathrm{RLR}+5.1\times \mathrm{NN}+13.7\times \mathrm{SVM}\right)}}$$

where RLR, NN, and SVM represent the probability scores obtained from the Ridge Logistic Regression (RLR), Neural Network (NN), and Support Vector Machine (SVM) models, respectively. Here, e denotes the base of the natural logarithm.

## 5. Conclusions

This study highlights the challenge of predicting nephrotoxicity using only physicochemical properties or off-target interactions. Although certain properties—such as the MDCK and Caco-2 cell permeability, PSA, fraction of *sp^3^* carbon atoms, and logD—differ between nephrotoxic and non-nephrotoxic drugs, these differences alone are insufficient for accurate predictions. The significant overlap between the two groups results in low sensitivity and specificity. However, when off-target interactions were included in the models, the prediction accuracy improved significantly. The combined model, using both physicochemical properties and off-target interactions, demonstrated better sensitivity, specificity, and predictive power (higher LR+). This approach is valuable in drug discovery, providing early indications of nephrotoxicity and helping to prioritize safer compounds. Still, validation with in vitro or ex vivo methods remains essential before advancing development candidate compounds to in vivo toxicology studies. Nonetheless, incorporating additional M-DIKI and N-DIKI chemical data in training sets, along with transcriptomics, into a unified framework will further increase the AI/ML models’ accuracy of DIKI predictions and offer an effective DIKI de-risking strategy early in the discovery process.

## Figures and Tables

**Table 1:** Computed physicochemical properties and corresponding *p*-values. Statistical analysis of physicochemical properties, with *p*-values < 0.05 indicating significance. Key properties such as permeability, PSA, fraction of sp^3^ carbon atoms, and logD show a significant association.

Physicochemical Property	*p*-Value
Total number of metabolites	0.18
Molecular weight	0.20
logS	0.36
Apparent MDCK cell permeability	≤0.001
Apparent Caco-2 cell permeability	≤0.001
PSA	≤0.001
pKa	0.30
Fraction of sp3-hybridized carbon atoms	0.01
logD	0.04

Abbreviations: MDCK (Madin–Darby canine kidney (MDCK) cells; PSA (polar surface area).

**Table 2:** Performance metrics for individual and combined ML models, comparing sensitivity, specificity, precision, NPV, accuracy, and AUC, using off-target interactions and physicochemical properties as features.

Method	Sensitivity (Recall)	Specificity	PPV (Precision)	NPV	Accuracy	AUC	LR+
RLR	0.77	0.78	0.66	0.85	0.77	0.87	3.42
NN	0.79	0.74	0.63	0.87	0.76	0.86	3.03
RF	0.83	0.70	0.61	0.88	0.74	0.82	2.72
SVM	0.82	0.72	0.62	0.87	0.75	0.73	2.90
RLR, NN	0.79	0.78	0.66	0.86	0.78	0.87	3.42
RLR, RF	0.77	0.72	0.61	0.85	0.74	0.83	2.76
RLR, SVM	0.78	0.78	0.66	0.87	0.78	0.87	3.43
NN, RF	0.77	0.75	0.64	0.86	0.76	0.84	3.13
NN, SVM	0.77	0.80	0.67	0.86	0.78	0.86	3.66
RF, SVM	0.77	0.72	0.60	0.85	0.73	0.82	2.71
RLR, NN, RF	0.79	0.75	0.64	0.86	0.76	0.84	3.13
RLR, NN, SVM	0.79	0.78	0.67	0.87	0.79	0.87	3.66
RLR, RF, SVM	0.77	0.75	0.64	0.85	0.75	0.83	3.08
NN, RF, SVM	0.79	0.75	0.65	0.87	0.77	0.84	3.29
RLR, NN, RF, SVM	0.79	0.76	0.65	0.86	0.776	0.77	3.3

Abbreviations: PPV (positive predictive value) and NPV (negative predictive value), AUC (area under the curve), RLR (Regularized Logistic Regression), NN (Neural Networks), RF (Random Forest), and SVM (Support Vector Machines).

**Table 3:** Performance metrics for individual and combined ML models, comparing sensitivity, specificity, precision, NPV, accuracy, and AUC, using off-target interactions only.

Method	Sensitivity (Recall)	Specificity	PPV (Precision)	NPV	Accuracy	AUC	LR+
RLR	0.87	0.65	0.59	0.90	0.73	0.86	2.53
NN	0.87	0.65	0.59	0.89	0.74	0.8 5	2.55
RF	0.97	0.28	0.43	0.95	0.53	0.79	1.34
SVM	0.87	0.62	0.57	0.91	0.72	0.82	2.38
RLR, NN	0.87	0.64	0.58	0.89	0.72	0.85	2.41
RLR, RF	0.87	0.64	0.58	0.90	0.72	0.85	2.43
RLR, SVM	0.87	0.65	0.59	0.90	0.73	0.86	2.51
NN, RF	0.87	0.64	0.57	0.89	0.71	0.85	2.36
NN, SVM	0.87	0.64	0.57	0.89	0.72	0.85	2.38
RF, SVM	0.56	0.87	0.71	0.78	0.76	0.81	4.30
RLR, NN, RF	0.87	0.64	0.57	0.89	0.72	0.85	2.38
RLR, NN, SVM	0.87	0.65	0.58	0.89	0.72	0.85	2.41
RLR, RF, SVM	0.87	0.64	0.58	0.89	0.72	0.85	2.41
NN, RF, SVM	0.87	0.62	0.56	0.89	0.70	0.84	2.27
RLR, NN, RF, SVM	0.85	0.64	0.57	0.89	0.71	0.84	2.36

Abbreviations: PPV (positive predictive value) and NPV (negative predictive value), AUC (area under the curve), RLR (Regularized Logistic Regression), NN (Neural Networks), RF (Random Forest), and SVM (Support Vector Machines).

**Table 4:** ML model performance metrics are compared using sensitivity, specificity, precision, NPV, accuracy, and AUC, with a focus on physicochemical properties only.

Method	Sensitivity (Recall)	Specificity	PPV (Precision)	NPV	Accuracy	AUC	LR+
RLR	0.59	0.65	0.48	0.74	0.62	0.62	1.66
NN	0.69	0.48	0.42	0.72	0.55	0.63	1.28
RF	0.67	0.49	0.42	0.73	0.55	0.73	1.28
SVM	0.69	0.47	0.42	0.73	0.54	0.70	1.27
RLR, NN	0.59	0.61	0.47	0.74	0.61	0.63	1.56
RLR, RF	0.71	0.64	0.52	0.79	0.66	0.73	1.94
RLR, SVM	0.67	0.67	0.52	0.78	0.66	0.70	1.95
NN, RF	0.72	0.64	0.53	0.80	0.67	0.73	1.96
NN, SVM	0.64	0.67	0.51	0.76	0.65	0.69	1.85
RF, SVM	0.72	0.64	0.53	0.80	0.67	0.73	1.97
RLR, NN, RF	0.72	0.64	0.53	0.79	0.67	0.73	1.96
RLR, NN, SVM	0.64	0.67	0.52	0.77	0.66	0.69	1.93
RLR, RF, SVM	0.72	0.64	0.53	0.80	0.67	0.73	1.98
NN, RF, SVM	0.72	0.64	0.53	0.79	0.67	0.73	1.98
RLR, NN, RF, SVM	0.72	0.64	0.53	0.80	0.67	0.73	1.97

Abbreviations: PPV (positive predictive value) and NPV (negative predictive value), AUC (area under the curve), RLR (Regularized Logistic Regression), NN (Neural Networks), RF (Random Forest), and SVM (Support Vector Machines).

**Table 5:** Performance metrics of best performing predictive models (RLR + NN + SVM) using combined off-target interactions and physicochemical descriptors compared to models using off-targets or physicochemical properties alone with integrated ML approaches.

Descriptors	Methods	Sensitivity	Specificity	PPV	NPV	Accuracy	AUC	LR+
Selected off-targets + selected physicochemical properties	RLR, NN, SVM	0.79	0.78	0.67	0.87	0.79	0.87	3.66
Selected off-targets	RLR, NN, SVM	0.87	0.65	0.58	0.89	0.72	0.85	2.41
All physicochemical properties	RLR, NN, SVM	0.64	0.67	0.52	0.77	0.66	0.69	1.93

## Data Availability

Where not included in the main text or Supplemental Materials, the data supporting the findings of this article may be available upon request from the corresponding author, Mohan Rao. Certain data are not publicly accessible due to restrictions, such as the inclusion of information that could compromise intellectual property. We developed the R package predDIKI, which includes a Shiny application for ease of use. The installation file and user manual are provided in the Supplementary Materials (README file). After installing the package, users can input chemical descriptors and biological interactions (e.g., gene names) for multiple small molecules to predict DIKI potential. The results include probability scores generated by various ML methods.

## Supplementary Materials

The following supporting information can be downloaded at https://www.mdpi.com/article/10.3390/ph17111550/s1. Supplemental Table S1: This Excel file compiles the predicted fifty-five physicochemical properties and DIKI classification for 360 compounds. Supplemental Table S2: This Excel file provides the predicted off-targets for 360 compounds. It includes the DIKI classification, compound names, predicted targets for each compound, whether the interaction is confirmed in vitro or predicted, and, for confirmed interactions, the reported activity in pIC50. Additionally, the file contains the predicted pIC50 for both confirmed and predicted interactions. Readme.pdf file provides installation instructions for the R-Shiny package zip file predDIKI_0.1.0.tar.gz.
