# Supplementary material for: Artificial Intelligence and Machine Learning Models for Predicting Drug-Induced Kidney Injury in Small Molecules
Source: Pharmaceuticals (Basel). 2024 Nov 19;17(11):1550. doi: 10.3390/ph17111550 (PMC11597314; doi:10.3390/ph17111550)
Supplement: Supplementary file 1 [file pharmaceuticals-17-01550-s001.zip › README.pdf]

READ ME

Artificial Intelligence and Machine Learning (AI/ML) Models for Predicting Drug-Induced Kidney Injury (DIKI) in Small Molecules

Mohan Rao, Vahid Nassiri, Sanjay Srivastava, Amy Yang, Satjit Brar, Eric McDuffie, and Clifford Sachs

Abstract

The predDIKI package predicts the probability of drug-induced kidney injury (DIKI) using key physicochemical properties and off-targets. This document explains how to install the package and use its Shiny application for easy DIKI risk prediction and classification.

Installation Instructions

Step 1: Install Required Packages

To use `predDIKI`, you first need to install the necessary R packages. The following R code will check and install any missing dependencies:

```r
# List of required packages
packages <- c(
        'openxlsx', 'ggplot2', 'reshape2', 'ggforce',
        'glmnet', 'caret', 'nnet', 'e1071',
        'ggrepel', 'shiny', 'testthat'
)

# Function to check and install missing packages
install_if_missing <- function(pkg) {
    if (!require(pkg, character.only = TRUE)) {
        install.packages(pkg, dependencies = TRUE)
        library(pkg, character.only = TRUE)
    }
}

# Install required packages
lapply(packages, install_if_missing)
```

Step 2: Install predDIKI Package

Once the required packages are installed, you can install the `predDIKI` package from a local file. Replace `'/path/to/predDIKI_0.1-0.tar.gz'` with the actual path to the package file:

```r
install.packages('/path/to/predDIKI_0.1-0.tar.gz', repos = NULL, type = 'source')
```

Step 3: Run the Shiny App

```
library(predDIKI)
predDIKI_app()
```

How to Use the Shiny Application

Step 1: Download the Input Template

- On the front page of the Shiny app (shown in **Figure 1**), click the "Download Template" button to get an Excel template.
- Fill in the required information about the physicochemical properties and off-targets for your test compounds.

Step 2: Upload Your Data

- Use the "Browse" button on the Shiny app to upload your completed Excel file.

Step 3: Run the Prediction

- After uploading the file, click the "Run predDIKI" button. The app will generate probability scores predicting nephrotoxicity for the test compounds.

Step 4: View and Download Results

- The results, including a violin plot comparing the nephrotoxicity probabilities for reference and test compounds, will be displayed (see **Figure 2**).
- Both the plot and the result table can be downloaded for further use.

Offline Use and Internal Deployment

Since the installation file and all source code are provided, `predDIKI` can be run entirely on your local machine without needing to upload data to a public server. If you want to deploy the application on an internal server, you can easily do so using free open-source technologies like ShinyProxy.

Figures

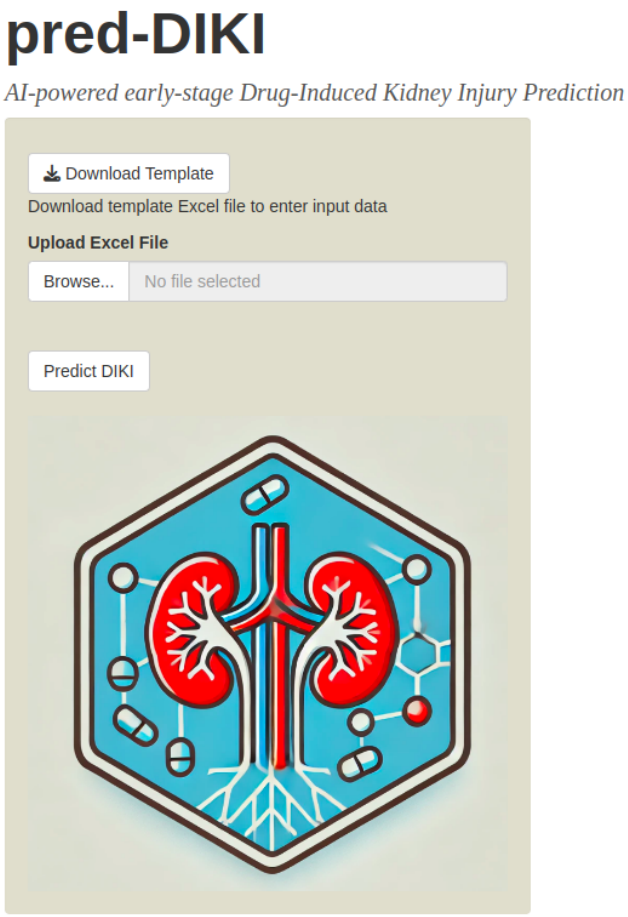

Figure 1: Front page of ‘predDIKI‘ Shiny application.

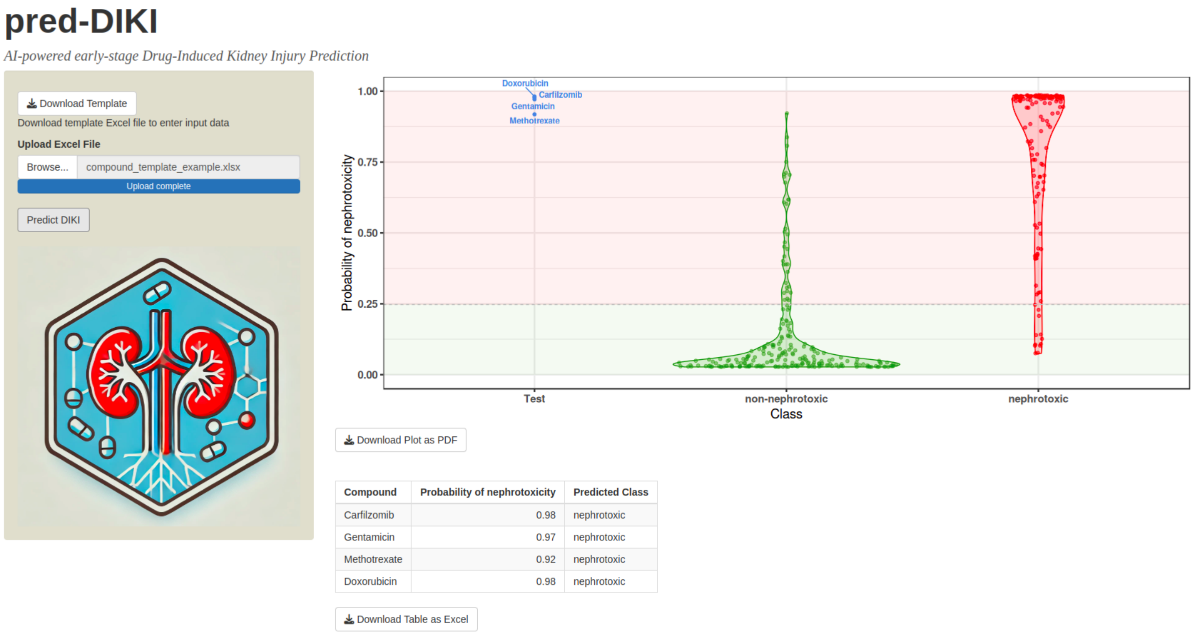

Figure 2: Example output from the ‘predDIKI‘ Shiny application.
